# Comparing transcranial direct current stimulation and transcranial random noise stimulation over left dorsolateral prefrontal cortex and left inferior frontal gyrus: Effects on divergent and convergent thinking

**DOI:** 10.3389/fnhum.2022.997445

**Published:** 2022-11-03

**Authors:** Javier Peña, Agurne Sampedro, Yolanda Balboa-Bandeira, Naroa Ibarretxe-Bilbao, Leire Zubiaurre-Elorza, M. Acebo García-Guerrero, Natalia Ojeda

**Affiliations:** Department of Psychology, Faculty of Health Sciences, University of Deusto, Bilbao, Spain

**Keywords:** transcranial direct current stimulation, transcranial random noise stimulation, divergent thinking, convergent thinking, dorsolateral prefrontal cortex, inferior frontal gyrus

## Abstract

The essential role of creativity has been highlighted in several human knowledge areas. Regarding the neural underpinnings of creativity, there is evidence about the role of left dorsolateral prefrontal cortex (DLPFC) and left inferior frontal gyrus (IFG) on divergent thinking (DT) and convergent thinking (CT). Transcranial stimulation studies suggest that the left DLPFC is associated with both DT and CT, whereas left IFG is more related to DT. However, none of the previous studies have targeted both hubs simultaneously and compared transcranial direct current stimulation (tDCS) and random noise stimulation (tRNS). Additionally, given the relationship between cognitive flexibility and creativity, we included it in order to check if the improvement in creativity may be mediated by cognitive flexibility. In this double-blind, between-subjects study, 66 healthy participants were randomly assigned to one of three groups (*N* = 22) that received a transcranial direct current stimulation (tDCS), transcranial random noise stimulation (tRNS), or sham for 20 min. The tDCS group received 1.5 mA with the anode over the left DLPFC and cathode over the left IFG. Locations in tRNS group were the same and they received 1.5 mA of high frequency tRNS (100–500 Hz). Divergent thinking was assessed before (baseline) and during stimulation with unusual uses (UU) and picture completion (PC) subtests from Torrance Creative thinking Test, whereas convergent thinking was evaluated with the remote association test (RAT). Stroop test was included to assess cognitive flexibility. ANCOVA results of performance under stimulation (controlling for baseline performance) showed that there were significant differences in PC (*F* = 3.35, *p* = 0.042, $n_{p}^{2}$ = 0.10) but not in UU (*F* = 0.61, *p* = 0.546) and RAT (*F* = 2.65, *p* = 0.079) scores. *Post-hoc* analyses showed that tRNS group had significantly higher scores compared to sham (*p* = 0.004) in PC. More specifically, tRNS showed higher performance in fluency (*p* = 0.012) and originality (*p* = 0.021) dimensions of PC compared to sham. Regarding cognitive flexibility, we did not find any significant effect of any of the stimulation groups (*F* = 0.34, *p* = 0.711). Therefore, no further mediation analyses were performed. Finally, the group that received tDCS reported more adverse effects than sham group (*F* = 3.46, *p* = 0.035). Altogether, these results suggest that tRNS may have some advantages over tDCS in DT.

## Introduction

The essential role of creativity has been highlighted in several areas, including science (Wilcox et al., 2018), mathematics (Mann, 2006), organizations (Ionescu et al., 2012), or economic and social development (Tremblay and Pilati, 2013) among others. Therefore, any attempt to promote it is a highly appreciated aim in science (Plucker et al., 2020).

According to several authors, there are two main components in creativity (Guilford, 1950; Cropley, 2006; Pick and Lavidor, 2019); convergent thinking (CT) and divergent thinking (DT). CT requires using deductive reasoning to find a single solution to a closed-ended problem (Zmigrod et al., 2015). According to many authors (Chermahini and Hommel, 2010, 2012; Dewhurst et al., 2011; Hommel et al., 2011; Colzato et al., 2013a,b, 2017; Sellaro et al., 2014; Mayseless and Shamay-Tsoory, 2015; Yamada and Nagai, 2015; Hoşgören Alici et al., 2019), the remote associates test (RAT) (Mednick, 1962) seems to be the most used instrument for CT assessment in the literature. However, although RAT seems to measure mainly CT, there is evidence that, to some extent, it may be reflecting also DT (Cortes et al., 2019).

Divergent thinking, on the other hand, is postulated as a type of thinking that produces multiple alternative and original responses to an open-ended problem (Guilford, 1967). Although many studies have used Alternative Uses Task (Guilford, 1967) or the Torrance Test of Creative Thinking (TTCT) (Torrance, 1966) to assess DT, similar to what has been previously mentioned with RAT, there is evidence suggesting that they also require CT processes (Cortes et al., 2019).

In recent years the interest to disentangle the neural underpinnings of creativity has increased (Weinberger et al., 2017). In this context, both neuroimaging studies neuroimaging (Gonen-Yaacovi et al., 2013; Beaty et al., 2015, 2016, 2019, 2021; Chrysikou, 2019; Sun et al., 2019) and transcranial stimulation studies have investigated this issue (Weinberger et al., 2017). A meta-analysis of neuroimaging studies (Boccia et al., 2015) suggests that the areas most related to DT tasks were the dorsolateral prefrontal cortex (DLPFC), inferior frontal gyrus (IFG), postcentral, and supramarginal gyri, inferior parietal lobule, insula, temporal gyrus (middle and superior), and middle occipital gyrus.

Results from transcranial stimulation studies mainly indicate that anodal tDCS over left DLPFC may increase DT (Colombo et al., 2015; Zmigrod et al., 2015; Peña et al., 2021) and CT (Cerruti and Schlaug, 2009; Metuki et al., 2012; Zmigrod et al., 2015; Peña et al., 2021, 2019). However, in separate studies, cathodal tDCS over the left IFG also found an increase in DT (Mayseless and Shamay-Tsoory, 2015; Kleinmintz et al., 2018; Hertenstein et al., 2019; Ivancovsky et al., 2019; Khalil et al., 2020; Chrysikou et al., 2021; Kenett et al., 2021).

Although less studied than tDCS, transcranial random noise stimulation (tRNS) in the high frequency range (100–500 Hz) has been used as a promising alternative to tDCS with the difference of being excitatory in both electrodes (Terney et al., 2008). Although the mechanisms for tRNS are not still completely understood, there are two main hypotheses. One hypothesis proposes that it induces a repetitive opening of the Na^+^ channels and therefore shortens the hyperpolarization phase (Terney et al., 2008; Chaieb et al., 2015). Another possible hypothesis suggests that the neuronal excitability increases through stochastic resonance, a phenomenon whereby the accumulation of random interference (i.e., noise) can increase the detection of weak stimuli or enhance the information content of a signal (Moss et al., 2004; Ward, 2009; Miniussi et al., 2013; van der Groen and Wenderoth, 2016).

Previous studies indicate that tRNS may provide additional advantages over tDCS. For example, a meta-analysis (Simonsmeier et al., 2018) showed that the effect of tRNS on language and mathematics was stronger compared to tDCS. Some authors have suggested that the effect of tRNS on visual perceptual learning (Terney et al., 2008) and working memory (Murphy et al., 2020) may be larger when compared to tDCS. Similarly, it is also suggested that tRNS may show longer term effects than tDCS (Snowball et al., 2013; Brevet-Aeby et al., 2019; Berger et al., 2021). Inukai et al. (2016) compared tDCS and tRNS for increasing cortical excitability and reported that tRNS produced the most significant increase. Vanneste et al. (2013) directly compared the response of patients with tinnitus after using tDCS, transcranial alternating current stimulation and tRNS techniques, showing that tRNS was superior to both tDCS and transcranial alternating current stimulation. Additionally, the possible adverse effects seem to be more tolerable after receiving tRNS (Fertonani et al., 2011). Moreover, tRNS is not as perceptible as tDCS regarding skin perception (Ambrus et al., 2010). In creativity, previous studies using only tRNS have shown a significant improvement in visual DT (originality) and RAT after posterior parietal cortex stimulation (Peña et al., 2020), and an improvement in RAT, unusual uses (fluency and originality) after stimulating DLPFC (Peña et al., 2019).

On the other hand, previous literature suggests that cognitive flexibility, measured with Stroop task, generally shows positive correlations with DT performance (Groborz and Necka, 2003; Edl et al., 2014; Xuejun et al., 2014; Sharma and Babu, 2017). Results from tDCS studies on cognitive flexibility suggest that the anodal stimulation of the DLPFC improves cognitive flexibility performance (Metuki et al., 2012; Dajani and Uddin, 2015; Borwick et al., 2020; Perrotta et al., 2021). However, previous studies have not tested if the effect of tDCS on creativity after targeting the DLPFC is partially due to the positive effect of tDCS on cognitive flexibility. In other words, we do not know if the improvement in cognitive flexibility after DLPFC partially mediates the impact on creativity enhancement.

The first objective of this study was to explore if tDCS with anode over the left DLPFC and cathode over the left IFG improves both CT and DT compared to sham. Additionally, we wanted to explore the effect of excitatory tRNS over the same brain areas. Given that the left DLPFC is related to cognitive control, we hypothesize that its stimulation would facilitate CT through the maintenance of focused attention and top-down support for relevant information (Fischer and Hommel, 2012; Zhang et al., 2020). Therefore, we hypothesize that both tDCS and tRNS groups will enhance CT due to the stimulation of left DLPFC compared to sham. Additionally, we hypothesize that tDCS group will relax top-down inhibitory constraints and improve bottom-up information processing due to the inhibition of left IFG with cathodal stimulation (Chrysikou, 2019), which in turn will improve DT compared to sham. The tRNS group, on the other hand, will have excitatory effects on both left DLPFC and left IFG. Finally, we hypothesize that the effect of tDCS and tRNS on creativity will be partially mediated by the enhancement in cognitive flexibility produced by the transcranial stimulation.

## Materials and methods

### Participants

We recruited 66 healthy and native Spanish speaking volunteers (aged 18 years or above) from the general population without restrictions on gender or handedness of participants. Participants fulfilled a screening questionnaire for transcranial electrical stimulation contra-indications that included: (1) previous history of brain surgery; (2) being pregnant; (3) suffering from frequent or severe headaches or migraines; (4) previous history or presence of neurological disorder or injury (epileptic or convulsive seizure, brain stroke, severe brain injury); and (5) presence of any brain metallic implant. The study obtained the ethical approval from the Research Ethics Committee of Deusto University (Ref: ETK-31/17-18). Participants did not receive any course credit or monetary or compensation for participating in the study. All volunteers provided written informed consent to participate in the study and they were free to withdraw at any time. All experimental procedures were conducted in accordance with the Declaration of Helsinki (2013).

### Design and procedure

**FIGURE 1:**
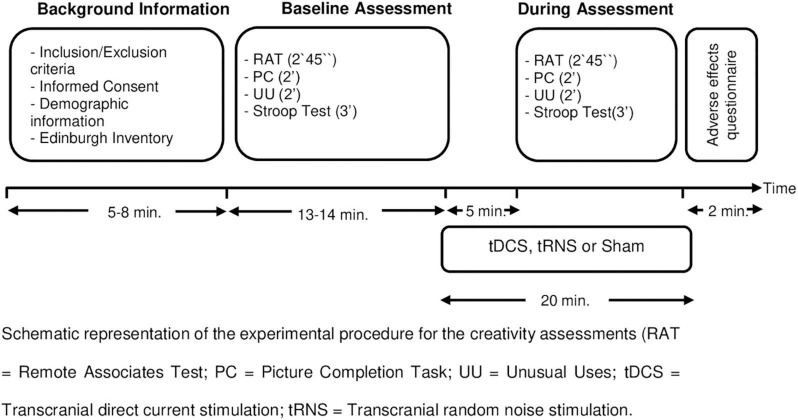
Study design. Schematic representation of the experimental procedure for the creativity assessments. RAT, remote associates test; PC, picture completion task; UU, unusual uses; tDCS, transcranial direct current stimulation; tRNS, transcranial random noise stimulation.

This was a double-blind, sham-controlled, parallel-group between-subjects design study and consisted of one single session. Figure 1 shows the study design and procedure. The participants were randomly assigned to one of the three groups (*n* = 22 in each group): tDCS group, tRNS group or sham group. Group assignment was based on a computer-generated randomization software.^1^ All raters were blind to stimulation group condition.

After signing the consent form, participants reported sociodemographic information along with tobacco consumption, hours of sleep and stimulant drinks ingested before the session.

The creativity assessment at baseline was carried out just before starting stimulation (see Figure 1). Participants were given 2 min and 45 s to complete the RAT and 2 min each for the UU and PC from the Torrance Test of Creative Thinking Test. Afterward, the participants underwent the Stroop test. They had 45 s for each Stroop condition (words, colors, and color-words).

Five minutes after the stimulation started, the participants were assessed with the parallel versions of RAT, UU, and PC with the same time limitations as the baseline assessment. The order of the version of UU, RAT, and PC were counterbalanced. Afterward, they completed again Stroop test. Participants filled the adverse effects questionnaire. In order to study the blinding efficacy, participants were asked to answer the following question: “Please, tell us if you think you were receiving real stimulation, no stimulation (placebo) or you do not know?”

### Application of transcranial direct current stimulation and transcranial random noise stimulation

Transcranial direct current stimulation and tRNS were delivered by using a wireless battery-operated Starstim8 device (Neuroelectrics Inc., Barcelona, Spain) attached to the back of a neoprene cap that follows the International 10–20 system. In the tDCS group, the anode was placed over the left DLPFC (F3 according to the 10/20 electrode placement EEG-System) and the cathode over the left posterior IFG (F7). In the tRNS group, the position of the electrodes (anodes) was the same as in tDCS group. Both real tDCS and tRNS groups received 20 min (with 30 s ramp up/down) of 1.5 mA tDCS or tRNS (high-frequency: 100–500 Hz) *via* two saline-soaked (5 ml per sponge) circular rubber-sponge electrodes (area of 8 cm^2^). The sham group received 30 s (with 30 s ramp-up/down) of real stimulation and kept the cap for 20 min as well. The impedance of the electrodes was checked before and during the stimulation to guarantee that it was maintained below 10 kΩ.

The stimulation protocol was created and monitored using the NIC 2.0 software.^2^ Stimulation groups were labeled as “Group A,” “Group B,” and “Group C” in the NIC2 software with the double-blind mode enabled. Therefore, the experimenters that applied the stimulation conditions were also blinded.

### Measures

#### The Torrance Test of Creative Thinking

We included unusual uses (UU) and picture completion (PC) subtests from *The Torrance Test of Creative Thinking* (Torrance, 1966). We included two different forms (Form A and B) for the baseline and during the stimulation assessments. We measured three dimensions for both UU and PC: fluency, originality, and flexibility.

In the PC task the participants are requested to complete ten unfinished figures by drawing additional elements in a paper and pencil task. Fluency was assessed as the total number of appropriate responses and the participants were given 1 point for each figure completed. The originality score was based on the statistical infrequency of each response based on the list of normative data (Torrance, 1966). They were given 1 point for each response considered original. Flexibility was assessed as the number of different ideational categories produced in the pictures, based on the list of categories from the Spanish adaptation of the TTCT (Jiménez et al., 2007). Fluency, originality and flexibility measures were converted to z-scores to obtain a PC composite. The internal consistency was good (Cronbach’s alpha = 0.76).

In the UU task, participants had to write down as many unusual uses as possible for an item. In the Form A of the test Cardboard Boxes was used as a stimulus. In the Form B Tin Cans was used. We measured three dimensions in UU: Fluency, Originality, and Flexibility. Fluency was obtained considering the number of different unusual uses produced (1 point for each response). Originality was based on the statistical unusualness of each response. We used the criteria based on the list of items from the manual (Torrance, 1966). A flexibility score was obtained from the number of different categories represented in the responses. Each different category was given 1 point. We also converted fluency, originality and flexibility measures to z-scores to obtain a UU composite. The internal consistency was good (Cronbach’s alpha = 0.86).

#### Remotes associates test

The Spanish version of the RAT (Mednick, 1962) was administered. Two different forms of the test were used for the baseline and during stimulation assessment. In RAT task, participants were asked to identify a word that is associated (either forming a compound word or semantically related) with three cue words. Each form included 30 items and participants had 2 min and 45 s for write down as many items as possible. The items were presented in the same sheet and participants could go backward and forward if they wished. The internal consistency of the test was high (Cronbach’s alpha = 0.81).

#### Stroop test

The Spanish version of the Stroop Test (Mednick, 1962; Golden, 2010) was administered. The Stroop test is a neuropsychological assessment tool composed of three parts: word, color, and word-color, each lasting 45 s. It is a widely used test for the assessment of processing speed (word subtest and color subtests) and cognitive flexibility (word-color and interference) in both clinical and healthy populations. It has a high internal consistency (Cronbach’s alpha = 0.80).

#### Questionnaire of adverse effects

A questionnaire with 11 items was used to measure any perceived side effects. We included numbness, tingling, skin redness, headache, itching sensation, concentration difficulties, burning, phosphenes, mood change, sore throat, and scalp pain study.

### Statistical analyses

Baseline characteristics were compared using the ANOVA test for continuous variables and *X*^2^ test for categorical data. Baseline correlation analyses were performed with Pearson’s R or Spearman’s Rho in case of non-normal distribution. Analysis of covariance (ANCOVA) was used to compare during stimulation scores (controlling for baseline scores) between the three groups for each of the creativity variables and Stroop (Wan, 2021). Effect size ($n_{p}^{2}$) was calculated for ANCOVA analyses. IBM SPSS software version 23.0 (IBM Corp. Released, 2015) was used for statistical analyses. All tests were two-tailed and the significance level was set at 0.05.

## Results

### Baseline characteristics of the groups

There were no significant differences between groups in any of the variables assessed at baseline (see Table 1). Participants were also asked to indicate if the number of drinks with stimulants ingested, number of hours slept, or number of tobacco consumption was less than usual, more than usual or the same as usual. There were no significant differences between the groups in sleeping hours [*X*^2^ (4, *N* = 66) = 6.46, *p* = 0.167], stimulant drinks [*X*^2^ (4, *N* = 66) = 3.41, *p* = 0.490], or tobacco consumption [*X*^2^ (4, *N* = 66) = 4.00, *p* = 0.406].

**TABLE 1:** Participant characteristics of transcranial direct current stimulation (tDCS), transcranial random noise stimulation (tRNS), and sham groups at baseline.

	tDCS	tRNS	Sham		
	Mean ± SD	Mean ± SD	Mean ± SD	Statistic	*P-value*
Age	31.86 ± 12.97	30.27 ± 13.09	28.50 ± 11.07	*F*(2,63) = 0.40	0.669
Years of education	13.50 ± 3.36	12.50 ± 2.84	11.86 ± 2.29	*F*(2,63) = 1.82	0.170
Gender: *n* (%)					
Females	12 (54.5%)	12 (54.5%)	11 (50.0%)	*X*^2^ (2, *N* = 66) = 0.12	0.941
Number of hours slept	6.91 ± 1.06	7.57 ± 1.21	7.27 ± 1.43	*F*(2,63) = 1.55	0.220
Edinburgh handedness	63.36 ± 51.24	65.30 ± 54.90	68.11 ± 37.89	*F*(2,56) = 0.16	0.850
Tobacco consumption	2.81 ± 3.05	1.54 ± 2.86	2.82 ± 4.23	*F*(2,63) = 1.00	0.371
Number of stimulants	1.04 ± 0.78	1.04 ± 0.95	1.09 ± 0.95	*F*(2,78) = 0.14	0.865

tDCS, transcranial direct current stimulation; tRNS, transcranial random noise stimulation; SD, standard deviation.

**FIGURE 2:**
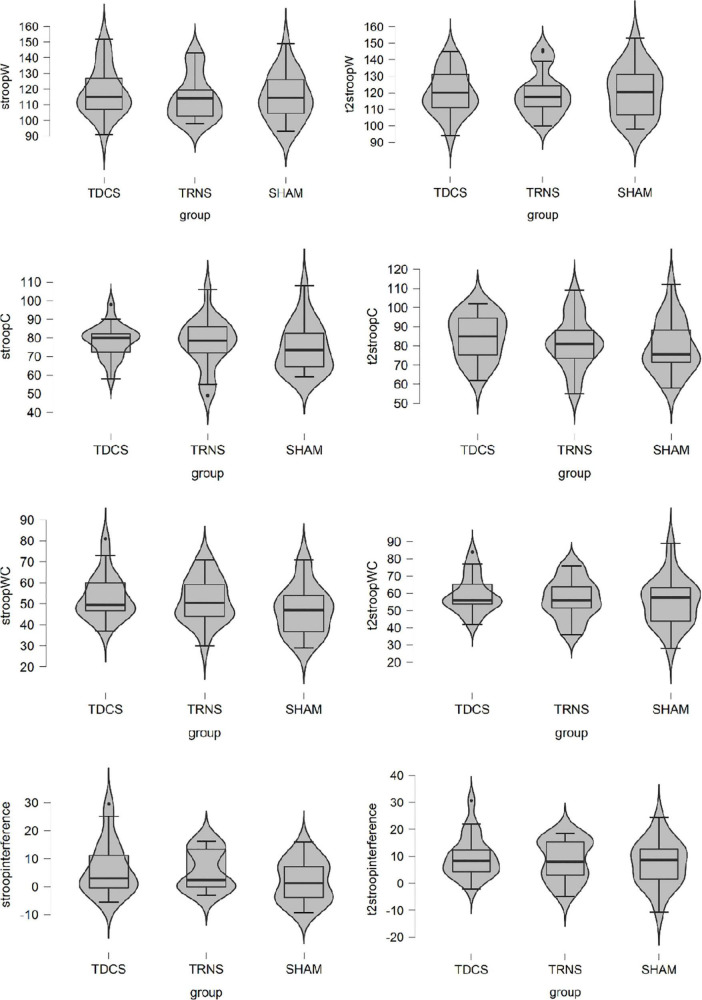
Stroop scores of transcranial direct current stimulation (tDCS), transcranial random noise stimulation (tRNS), and sham group at baseline and during stimulation.

The RAT, UU, and PC scores of tDCS, tRNS, and sham groups at baseline and during stimulation are shown in Table 2 whereas the scores in Stroop Test are shown in Figure 2.

**TABLE 2:** Creativity scores of transcranial direct current stimulation (tDCS), transcranial random noise stimulation (tRNS), and sham groups at baseline and during stimulation.

		tDCS	tRNS	Sham
		Mean ± SD	Mean ± SD	Mean ± SD
RAT	Baseline	6.59 ± 3.06	6.59 ± 2.85	7.31 ± 2.81
	During	7.86 ± 2.96	7.63 ± 3.27	6.59 ± 3.19
UU fluency	Baseline	7.86 ± 3.22	7.22 ± 2.20	6.72 ± 2.25
	During	8.09 ± 3.78	7.72 ± 2.45	6.54 ± 2.90
UU originality	Baseline	4.13 ± 2.64	3.09 ± 2.16	3.04 ± 1.91
	During	4.22 ± 3.28	4.18 ± 2.28	3.54 ± 2.17
UU flexibility	Baseline	5.59 ± 2.34	5.50 ± 1.79	5.36 ± 1.33
	During	5.90 ± 2.70	5.86 ± 1.55	5.27 ± 2.37
PC fluency	Baseline	6.00 ± 1.77	5.36 ± 1.70	5.13 ± 1.91
	During	6.59 ± 1.96	6.54 ± 1.76	5.31 ± 1.67
PC originality	Baseline	2.13 ± 1.32	1.95 ± 1.55	2.00 ± 1.63
	During	2.22 ± 1.34	2.77 ± 1.63	1.72 ± 1.38
PC flexibility	Baseline	5.27 ± 1.51	5.00 ± 1.51	4.45 ± 1.50
	During	5.86 ± 1.58	6.04 ± 1.70	5.09 ± 2.04

tDCS, transcranial direct current stimulation; tRNS, transcranial random noise stimulation; SD, standard deviation; Verbal RAT, number of correct answers in remote associates test; PC, picture completion from Torrance Test of Creative Thinking; UU, unusual uses from Torrance Test of Creative Thinking.

### Stimulation effects on remote association test, unusual uses, and picture completion

The ANCOVA results are shown in Table 3. RAT scores did not reach a statistically significant results in overall ANCOVA (*F* = 2.65, *p* = 0.079). Exploratory *post-hoc* analyses indicated that the comparison between tDCS and sham group was significant (*p* = 0.038) and between tRNS and sham was marginally significant (*p* = 0.071). We must take these exploratory results with cautious, since the overall ANCOVA was not significant.

**TABLE 3:** Differences among groups in remote association test (RAT), unusual uses (UU), and picture completion (PC) scores during stimulation after controlling for baseline scores.

	tDCS	tRNS	Sham					
	Marginal Mean ± SE	Marginal Mean ± SE	Marginal Mean ± SE	*F*	*p*	${\text{n}}_{p}^{2}$	Comparison group	*Post-hoc P-value*
RAT	8.01 ± 0.56	7.78 ± 0.56	6.30 ± 0.56	2.64	0.079	0.07	tDCS vs. tRNS	0.777
							tDCS vs. Sham	**0.038**
							tRNS vs. Sham	0.071
UU fluency	7.59 ± 0.47	7.76 ± 0.47	7.00 ± 0.47	0.71	0.495	0.02	tDCS vs. tRNS	0.796
							tDCS vs. Sham	0.387
							tRNS vs. Sham	0.258
UU originality	3.78 ± 0.48	4.38 ± 0.48	3.78 ± 0.48	0.52	0.593	0.01	tDCS vs. tRNS	0.387
							tDCS vs. Sham	0.990
							tRNS vs. Sham	0.372
UU flexibility	5.82 ± 0.37	5.85 ± 0.37	5.36 ± 0.37	0.53	0.081	0.01	tDCS vs. tRNS	0.964
							tDCS vs. Sham	0.390
							tRNS vs. Sham	0.365
PC fluency	6.26 ± 0.29	6.63 ± 0.29	5.55 ± 0.29	3.44	**0.038**	0.10	tDCS vs. tRNS	0.379
							tDCS vs. Sham	0.101
							tRNS vs. Sham	**0.012**
PC originality	2.18 ± 0.28	2.80 ± 0.28	1.74 ± 0.28	3.58	**0.034**	0.10	tDCS vs. tRNS	0.125
							tDCS vs. Sham	0.272
							tRNS vs. Sham	**0.010**
PC flexibility	5.60 ± 0.31	5.98 ± 0.30	5.41 ± 0.31	0.87	0.424	0.02	tDCS vs. tRNS	0.393
							tDCS vs. Sham	0.667
							tRNS vs. Sham	0.202

tDCS, transcranial direct current stimulation; tRNS, transcranial random noise stimulation; SE, standard error; PC, picture completion from Torrance Test of Creative Thinking; UU, unusual uses from Torrance Test of Creative Thinking; $n_{\text{p}}^{2}$, Eta partial Squared. Bold values represent the statistically signifcant.

Regarding PC subdomains, we found significant differences among the groups in PC fluency (*F* = 3.35, *p* = 0.042) and originality (*F* = 3.35, *p* = 0.042). *Post-hoc* analyses revealed that tRNS group scored significantly higher than sham on fluency (*p* = 0.012) and originality (*p* = 0.010) whereas there were no significant differences among the rest of comparisons. Finally, the composite PC score was significantly different between groups (*F* = 3.35, *p* = 0.042). *Post-hoc* analyses indicated that the tRNS group (0.27 ± 0.11 standard error) scored significantly higher than sham group (−0.26 ± 0.14 standard error, *p* = 0.012) but not higher than tDCS group (−0.02 ± 0.15 standard error, *p* = 0.162).

Regarding UU scores, contrary to expected, we did not find any significant difference in any of the domains analyzed, nor in the UU composite score.

Based on the fluency scores, we calculated the percentage of original responses (number of original responses × 100/total number of correct responses) for both PC and UU. Although the number of original responses was significant for PC, there were not significant differences in the percentage of original responses in PC [*F*(2,62) = 1.72, *p* = 0.188]. Similarly, results in the percentage of original responses in UU showed no significant differences [*F*(2,62) = 0.42, *p* = 0.658].

### Stimulation effects on Stroop test

We did not find any significant effect on any of the measures of Stroop Test (see Table 4). Therefore, we could not further test if the improvement in creative performance was partially due to an improvement in cognitive flexibility.

**TABLE 4:** Differences among groups in Stroop scores during stimulation after controlling for baseline scores.

	tDCS	tRNS	Sham					
	Marginal Mean ± SE	Marginal Mean ± SE	Marginal Mean ± SE	*F*	*p*	${\text{n}}_{p}^{2}$	Comparison group	*Post-hoc P-value*
Stroop-W	118.14 ± 1.39	120.86 ± 1.39	121.34 ± 1.39	1.51	0.23	0.05	tDCS vs. tRNS	0.176
							tDCS vs. Sham	0.112
							tRNS vs. Sham	0.81
Stroop-C	82.94 ± 1.49	81.02 ± 1.49	81.53 ± 1.49	0.44	0.645	0.01	tDCS vs. tRNS	0.367
							tDCS vs. Sham	0.51
							tRNS vs. Sham	0.811
Stroop-WC	56.67 ± 1.44	55.69 ± 1.43	58.63 ± 1.45	1.06	0.354	0.03	tDCS vs. tRNS	0.629
							tDCS vs. Sham	0.348
							tRNS vs. Sham	0.157
Stroop Interference	8.66 ± 1.31	7.70 ± 1.30	9.23 ± 1.32	0.34	0.711	0.06	tDCS vs. tRNS	0.603
							tDCS vs. Sham	0.765
							tRNS vs. Sham	0.419

Stroop-W, Stroop word subtest from Stroop; Stroop-C, Stroop color subtest from Stroop; Stroop-WC, Stroop word-color subtest from Stroop; tDCS, transcranial direct current stimulation; tRNS, transcranial random noise stimulation; SE, standard error; $n_{\text{p}}^{2}$, Eta partial Squared.

### Correlation analyses between Stroop performance and creativity measures at baseline

Table 5 shows the correlation analyses between Stroop test and creativity scores at baseline in the whole sample. UU dimension of fluency, originality, and flexibility were significantly correlated with Stroop-W, Stroop-C, and Stroop-WC subtests, whereas interference score did not correlate with any of the creativity measures included.

**TABLE 5:** Baseline correlations between Stroop scores and divergent thinking (DT) and convergent thinking (CT) tasks.

	RAT	PC fluency	PC originality	PC flexibility	UU fluency	UU originality	UU flexibility
Stroop-W	0.31 ρ*	0.09 ρ	0.16 ρ	0.10 ρ	0.36 ρ**	0.33 ρ**	0.38 ρ**
Stroop-C	0.05	−0.07	0.14 ρ	−0.02 ρ	0.39**	0.29 ρ*	0.40 ρ**
Stroop-WC	0.11	−0.04	0.07 ρ	0.16 ρ	0.30*	0.35 ρ**	0.35 ρ**
Stroop-interference	0.03 ρ	0.00 ρ	−0.05 ρ	0.15 ρ	0.04 ρ	0.23 ρ	0.13 ρ

Stroop-W, Stroop word subtest from Stroop; Stroop-C, Stroop color subtest from Stroop; Stroop-WC, Stroop word-color subtest from Stroop; RAT, number of correct answers in remote associates test; PC, picture completion from Torrance Test of Creative Thinking; UU, unusual uses from Torrance Test of Creative Thinking; ρ, Spearman’s Rho. *p < 0.05, **p < 0.001.

### Adverse effects and blinding

None of the participants reported having experienced any significant adverse effects that made them leave the study. There were significant differences among the groups in minor adverse effects [*F*(2,62) = 3.46, *p* = 0.035]. *Post-hoc* analyses indicated that tDCS group (*M* = 2.18, SD = 1.70) reported more adverse effects than tRNS (*M* = 1.29, SD = 1.34, *p* = 0.046) and sham groups (*M* = 1.29, SD = 1.34, *p* = 0.015). We additionally investigated if the total number of adverse effects was correlated with the change in creative scores. However, we did not find any significant effect of adverse effects in any of the creativity changes analyzed nor in the whole sample (*p*-values ranged from 0.193 to 0.970) neither in the stimulation groups (*p*-values ranged from 0.133 to 0.909).

We did not find significant differences in stimulation guess between real and sham conditions [χ^2^ (2, *N* = 66) = 5.78, *p* = 0.216]. From the tDCS group, 36.4% guessed that they had received stimulation, 22.7% guessed they had received the placebo and 40.9% were undecided. From the tRNS group, 9.1% guessed that they had received stimulation, 40.9% guessed they had received the placebo, and 50.0% were undecided. Finally, from the sham group, 34.8% guessed that they had received the placebo, 27.3% that they had received stimulation, and 33.3% were undecided. We did not find any significant difference in creative performance changes between those who guessed they were receiving real stimulation compared to those guessing they received sham or they did not know (*p*-values ranged from 0.181 to 0.990).

## Discussion

This study compared the effect of tDCS and tRNS over the left DLPFC and left IFG simultaneously on DT and CT performance. The results showed that fluency and originality dimensions of visual DT benefited from tRNS stimulation compared to sham, whereas contrary to expected, tDCS did not show any significant effect on DT. The positive effect of tRNS over the left DLPFC on the fluency dimension of DT is to some extent consistent with previous literature (Lustenberger et al., 2015; Zmigrod et al., 2015; Grabner et al., 2018; Peña et al., 2019, 2021). However, the significant effect of tRNS on originality was not expected since some of the previous studies have shown even a reduction in novelty of responses after the excitatory stimulation of the left IFG, which was what the tRNS group received (Ivancovsky et al., 2019; Kenett et al., 2021). We initially hypothesized that the anodal stimulation with tDCS over the left DLPFC with the simultaneous cathodal stimulation over the left IFG would enhance both verbal and visual DT more than the stimulation of both DLPFC and IFG with tRNS, since previous studies have shown independently that the stimulation of the left DLPFC is associated with DT (Colombo et al., 2015; Zmigrod et al., 2015) and separate studies using cathodal stimulation over the left IFG have also shown an improvement in DT (Chrysikou et al., 2013; Mayseless and Shamay-Tsoory, 2015; Hertenstein et al., 2019; Ivancovsky et al., 2019; Khalil et al., 2020). However, the current results suggest that it was tRNS and not tDCS that enhanced DT. A possible reason for this result may be that the simultaneous stimulation of both left DLPFC and IFG with tRNS may have had a positive on DT by enhancing one×s thoughts toward a specific aim under an increase in selection demands, possibly mediated by the left DLPFC (Peña et al., 2019). The left DLPFC has been related to executive functioning and working memory (Andrews et al., 2011), which in turn affect cognitive control (Andrews et al., 2011). Therefore, the stimulation of left DLPFC may enhance DT through the maintenance of focused-attention and inhibiting task-irrelevant information (Beaty and Schacter, 2018). An additional evidence for this possible explanation comes from the results obtained in the percentage of original responses, which showed not significant results. Therefore, the main effect of tRNS may have been on the fluency dimension, which in turn may have produced more total original ideas, but not a higher proportion of original ideas.

Regarding CT, our results were only marginally significant and it may mean a lack of statistical power, so we cannot conclude that tDCS neither tRNS over left DLPFC and IFG do significantly affect RAT scores. However, the trend toward a better performance after both tDCS and tRNS would be consistent with many studies that have shown that the stimulation of the left DLPFC is associated with an improvement in RAT using both tDCS (Cerruti and Schlaug, 2009; Zmigrod et al., 2015) and tRNS (Peña et al., 2019, 2021). A possible explanation for this result is that the stimulation of left DLPFC would enhance RAT performance by strengthening the maintenance of focused attention, top-down support for relevant information, manipulation of information in working memory and inhibition of task-irrelevant information (Fischer and Hommel, 2012; Zhang et al., 2020).

Further exploratory analyses found that cognitive flexibility, measured with Stroop, positively correlated with verbal DT score and its three subdimensions (fluency, originality, and flexibility). This result is consistent with previous literature (Groborz and Necka, 2003; Edl et al., 2014; Xuejun et al., 2014; Sharma and Babu, 2017). However, contrary to expected, we did not find any significant effect of tDCS or tRNS on cognitive flexibility measured with the Stroop Test. Therefore, we could not show that the effect of brain stimulation over the left DLPFC and IFG on creative performance was partially mediated by the enhancement of cognitive flexibility, which in turn would enhance creativity. Previous studies using tDCS over the DLPFC have found a significant effect, revealing the prominent role of DLPFC on cognitive flexibility measured with the Stroop Test (Metuki et al., 2012; Dajani and Uddin, 2015; Borwick et al., 2020; Perrotta et al., 2021).

Our results indicate that tRNS produced less adverse effects compared to tDCS. These results are consistent with previous studies that found that tRNS-induced sensations were less frequently perceived compared to tDCS (Poreisz et al., 2007; Ambrus et al., 2010), especially with itching irritation and burning sensations (Fertonani et al., 2011). Based on these results, some authors suggest that tRNS may be an optimal tool for experimental designs (Fertonani et al., 2011).

Although the interesting results, there are several limitations in this study. Firstly, we included only one item per time point for both UU and PC, which may explain the lack of significant results in verbal DT. Additionally, the RAT measure included mostly associative items but also compound items. Another limitation is that since high frequency tRNS is mainly excitatory, we could not completely compare the effect of tDCS and tRNS, since it is expected to affect in a different way over the left IFG. In other words, the excitatory effect over the left DLPFC is supposed to be similar. However, the cathodal tDCS over the left IFG is suggested to exert an inhibitory effect whereas tRNS is expected to be excitatory. Similarly, we only included two electrodes in the montage. Given the complex nature of creativity, future studies should use multichannel stimulation in order to stimulate cerebral networks instead of discrete brain areas.

## Data availability statement

The raw data supporting the conclusions of this article will be made available by the authors, without undue reservation.

## Ethics statement

The studies involving human participants were reviewed and approved by Research Ethics Committee of Deusto University (Ref: ETK-31/17-18). The patients/participants provided their written informed consent to participate in this study.

## Author contributions

JP: conceptualization, planning, data collection, data analysis, and writing of the manuscript. AS: conceptualization, data collection, supporting data analysis, and proofreading. YB-B and NO: supporting data analysis and proofreading. NI-B: data collection and proofreading. LZ-E: supporting data collection and proofreading. MG-G: conceptualization and supporting data analysis. All authors contributed to the article and approved the submitted version.

## Footnotes

1
www.randomizer.org

2
www.neuroelectrics.com/products/software/nic2/

## References

[B1] Ambrus G. G. Paulus W. Antal A. (2010). Cutaneous perception thresholds of electrical stimulation methods: Comparison of tDCS and tRNS. *Clin. Neurophysiol*. 121 1908–1914. 10.1016/j.clinph.2010.04.020 20471313

[B2] Andrews S. C. Hoy K. E. Enticott P. G. Daskalakis Z. J. Fitzgerald P. B. (2011). Improving working memory: The effect of combining cognitive activity and anodal transcranial direct current stimulation to the left dorsolateral prefrontal cortex. *Brain Stimul.* 4 84–89. 10.1016/j.brs.2010.06.004 21511208

[B3] Beaty R. E. Benedek M. Kaufman S. B. Silvia P. J. (2015). Default and executive network coupling supports creative idea production. *Sci. Rep.* 5:10964. 10.1038/srep10964 26084037 PMC4472024

[B4] Beaty R. E. Benedek M. Silvia P. J. Schacter D. L. (2016). Creative cognition and brain network dynamics. *Trends Cogn. Sci.* 20 87–95. 10.1016/j.tics.2015.10.004 26553223 PMC4724474

[B5] Beaty R. E. Seli P. Schacter D. L. (2019). Network neuroscience of creative cognition: Mapping cognitive mechanisms and individual differences in the creative brain. *Curr. Opin. Behav. Sci.* 27 22–30. 10.1016/j.cobeha.2018.08.013 30906824 PMC6428436

[B6] Beaty R. Schacter D. (2018). “Episodic memory and cognitive control: Contributions to creative idea production,” in *The cambridge handbook of the neuroscience of creativity*, (Cambridge: Cambridge University Press), 249.

[B7] Beaty R. Cortes R. A. Zeitlen D. C. Weinberger A. B. Green A. E. (2021). Functional realignment of frontoparietal subnetworks during divergent creative thinking. *Cereb. Cortex* 31 4464–4476. 10.1093/cercor/bhab100 33895837

[B8] Berger I. Dakwar-Kawar O. Grossman E. S. Nahum M. Cohen Kadosh R. (2021). Scaffolding the attention-deficit/hyperactivity disorder brain using transcranial direct current and random noise stimulation: A randomized controlled trial. *Clin. Neurophysiol.* 132 699–707. 10.1016/j.clinph.2021.01.005 33561725

[B9] Boccia M. Piccardi L. Palermo L. Nori R. Palmiero M. (2015). Where do bright ideas occur in ourbrain? Meta-analytic evidence from neuroimaging studies of domain-specific creativity. *Front. Psychol.* 6:1195. 10.3389/fpsyg.2015.01195 26322002 PMC4531218

[B10] Borwick C. Lal R. Lim L. W. Stagg C. J. Aquili L. (2020). Dopamine depletion effects on cognitive flexibility as modulated by tDCS of the dlPFC. *Brain Stimul.* 13 105–108. 31494070 10.1016/j.brs.2019.08.016 PMC7116421

[B11] Brevet-Aeby C. Mondino M. Poulet E. Brunelin J. (2019). Three repeated sessions of transcranial random noise stimulation (tRNS) leads to long-term effects on reaction time in the Go/No Go task. *Neurophysiol. Clin.* 49 27–32. 10.1016/j.neucli.2018.10.066 30414823

[B12] Cerruti C. Schlaug G. (2009). Anodal transcranial direct current stimulation of the prefrontal cortex enhances complex verbal associative thought. *J. Cogn. Neurosci.* 21 1980–1987. 10.1162/jocn.2008.21143 18855556 PMC3005595

[B13] Chaieb L. Antal A. Paulus W. (2015). Transcranial random noise stimulation-induced plasticity is NMDA-receptor independent but sodium-channel blocker and benzodiazepines sensitive. *Front. Neurosci.* 9:125. 10.3389/fnins.2015.00125 25914617 PMC4392589

[B14] Chermahini S. A. Hommel B. (2010). The (b)link between creativity and dopamine: Spontaneous eye blink rates predict and dissociate divergent and convergent thinking. *Cognition* 115 458–465. 10.1016/j.cognition.2010.03.007 20334856

[B15] Chermahini S. A. Hommel B. (2012). Creative mood swings: Divergent and convergent thinking affect mood in opposite ways. *Psychol. Res.* 76 634–640. 10.1007/s00426-011-0358-z 21695470 PMC3412079

[B16] Chrysikou E. G. (2019). Creativity in and out of (cognitive) control. *Curr. Opin. Behav. Sci.* 27 94–99. 10.1016/j.cobeha.2018.09.014

[B17] Chrysikou E. G. Hamilton R. H. Coslett H. B. Datta A. Bikson M. Thompson-Schill S. L. (2013). Noninvasive transcranial direct current stimulation over the left prefrontal cortex facilitates cognitive flexibility in tool use. *Cogn. Neurosci.* 4 81–89. 10.1080/17588928.2013.768221 23894253 PMC3719984

[B18] Chrysikou E. G. Morrow H. M. Flohrschutz A. Denney L. (2021). Augmenting ideational fluency in a creativity task across multiple transcranial direct current stimulation montages. *Sci. Rep.* 11:8874. 10.1038/s41598-021-85804-3 33893329 PMC8065129

[B19] Colombo B. Bartesaghi N. Simonelli L. Antonietti A. (2015). The combined effects of neurostimulation and priming on creative thinking. A preliminary tDCS study on dorsolateral prefrontal cortex. *Front. Hum. Neurosci.* 9:403. 10.3389/fnhum.2015.00403 26236219 PMC4505103

[B20] Colzato L. S. Szapora A. Lippelt D. Hommel B. (2017). Prior meditation practice modulates performance and strategy use in convergent- and divergent-thinking problems. *Mindfulness* 7 152–159. 10.1007/s12671-014-0352-9

[B21] Colzato L. S. Szapora A. Pannekoek J. N. Hommel B. (2013a). The impact of physical exercise on convergent and divergent thinking. *Front. Hum. Neurosci.* 7:824. 10.3389/fnhum.2013.00824 24348370 PMC3845014

[B22] Colzato L. S. van den Wildenberg W. P. M. Hommel B. (2013b). Increasing self-other integration through divergent thinking. *Psychon. Bull. Rev.* 20 1011–1016. 10.3758/s13423-013-0413-4 23440727

[B23] Cortes R. A. Weinberger A. B. Daker R. J. Green A. E. (2019). Re-examining prominent measures of divergent and convergent creativity. *Curr. Opin. Behav. Sci.* 27 90–93. 10.1016/j.cobeha.2018.09.017

[B24] Cropley A. (2006). In praise of convergent thinking. *Creat. Res. J.* 18 391–404. 10.1207/s15326934crj1803_13

[B25] Dajani D. R. Uddin L. Q. (2015). Demystifying cognitive flexibility: Implications for clinical and developmental neuroscience. *Trends Neurosci.* 38 571–578. 10.1016/j.tins.2015.07.003 26343956 PMC5414037

[B26] Dewhurst S. A. Thorley C. Hammond E. R. Ormerod T. C. (2011). Convergent, but not divergent, thinking predicts susceptibility to associative memory illusions. *Pers. Ind. Differ.* 51 73–76. 10.1016/j.paid.2011.03.018

[B27] Edl S. Benedek M. Papousek I. Weiss E. M. Fink A. (2014). Creativity and the stroop interference effect. *Pers. Ind. Differ.* 69 38–42. 10.1016/j.paid.2014.05.009

[B28] Fertonani A. Pirulli C. Miniussi C. (2011). Random noise stimulation improves neuroplasticity in perceptual learning. *J. Neurosci.* 31 15416–15423. 10.1523/jneurosci.2002-11.2011 22031888 PMC6703532

[B29] Fischer R. Hommel B. (2012). Deep thinking increases task-set shielding and reduces shifting flexibility in dual-task performance. *Cognition* 123 303–307. 10.1016/j.cognition.2011.11.015 22336726

[B30] Golden C. J. (2010). *STROOP. Test de colores y palabras*, 5 Edn. Madrid: TEA Ediciones.

[B31] Gonen-Yaacovi G. de Souza L. C. Levy R. Urbanski M. Josse G. Volle E. (2013). Rostral and caudal prefrontal contribution to creativity: A meta-analysis of functional imaging data. *Front. Hum. Neurosci.* 7:465. 10.3389/FNHUM.2013.00465 23966927 PMC3743130

[B32] Grabner R. H. Krenn J. Fink A. Arendasy M. Benedek M. (2018). Effects of alpha and gamma transcranial alternating current stimulation (tACS) on verbal creativity and intelligence test performance. *Neuropsychologia* 118 91–98. 10.1016/j.neuropsychologia.2017.10.035 29100950

[B33] Groborz M. Necka E. (2003). Creativity and cognitive control: Explorations of generation and evaluation skills. *Creat. Res. J.* 15 183–197. 10.1080/10400419.2003.9651411

[B34] Guilford J. P. (1950). Creativity. *Am. Psychol.* 5 444–454. 10.1037/h0063487 14771441

[B35] Guilford J. P. (1967). *The nature of human intelligence.* New York, NY: McGraw Hill, 10.4018/978-1-59904-426-2.ch007

[B36] Hertenstein E. Waibel E. Frase L. Riemann D. Feige B. Nitsche M. A. (2019). Modulation of creativity by transcranial direct current stimulation. *Brain Stimul.* 12 1213–1221. 10.1016/j.brs.2019.06.004 31231043

[B37] Hommel B. Colzato L. S. Fischer R. Christoffels I. K. (2011). Bilingualism and creativity: Benefits in convergent thinking come with losses in divergent thinking. *Front. Psychol.* 2:273. 10.3389/fpsyg.2011.00273 22084634 PMC3212749

[B38] Hoşgören Alici Y. Özgüven H. D. Kale E. Yenihayat I. Baskak B. (2019). Prefrontal activity measured by functional near infrared spectroscopy during divergent and convergent thinking in bipolar disorder. *Noropsikiyatri Ars.* 56 86–91. 10.29399/npa.23203 31223238 PMC6563865

[B39] IBM Corp. Released (2015). *IBM SPSS Statistics for Windows, Version 23.0, 2015.*

[B40] Inukai Y. Saito K. Sasaki R. Tsuiki S. Miyaguchi S. Kojima S. (2016). Comparison of three non-invasive transcranial electrical stimulation methods for increasing cortical excitability. *Front. Hum. Neurosci.* 10:668. 10.3389/fnhum.2016.00668 28082887 PMC5186778

[B41] Ionescu V. Cornescu V. Druic E. (2012). Creativity, innovation and change in knowledge-based organization. *Rev. Econ.* 2, 160–166.

[B42] Ivancovsky T. Kurman J. Morio H. Shamay-Tsoory S. (2019). Transcranial direct current stimulation (tDCS) targeting the left inferior frontal gyrus: Effects on creativity across cultures. *Soc. Neurosci.* 14 277–285. 10.1080/17470919.2018.1464505 29641936

[B43] Jiménez J. E. Artiles C. Rodríguez Cy García E. (2007). *Adaptación y baremación del test de pensamiento creativo de Torrance: Expresión figurada.* Canarias: Consejería de Educación.

[B44] Kenett Y. N. Tamez E. R. Thompson-Schill S. L. (2021). Noninvasive brain stimulation to lateral prefrontal cortex alters the novelty of creative idea generation. *Cogn. Affect. Behav. Neurosci.* 21 311–326. 10.3758/S13415-021-00869-X 33624232 PMC8122037

[B45] Khalil R. Karim A. A. Kondinska A. Godde B. (2020). Effects of transcranial direct current stimulation of left and right inferior frontal gyrus on creative divergent thinking are moderated by changes in inhibition control. *Brain Struct. Funct.* 225 1691–1704. 32556475 10.1007/s00429-020-02081-y PMC7321900

[B46] Kleinmintz O. M. Abecasis D. Tauber A. Geva A. Chistyakov A. V. Kreinin I. (2018). Participation of the left inferior frontal gyrus in human originality. *Brain Struct. Funct.* 223 329–341. 10.1007/s00429-017-1500-5 28828749

[B47] Lustenberger C. Boyle M. R. Foulser A. A. Mellin J. M. Fröhlich F. (2015). Functional role of frontal alpha oscillations in creativity. *Cortex* 67 74–82. 10.1016/j.cortex.2015.03.012 25913062 PMC4451406

[B48] Mann E. L. (2006). Creativity: The essence of mathematics. *J. Educ. Gifted* 30 236–260. 10.4219/jeg-2006-264

[B49] Mayseless N. Shamay-Tsoory S. G. (2015). Enhancing verbal creativity: Modulating creativity by altering the balance between right and left inferior frontal gyrus with tDCS. *Neuroscience* 291 167–176. 10.1016/j.neuroscience.2015.01.061 25659343

[B50] Mednick S. (1962). The associative basis of the creative process. *Psychol. Rev.* 69 220–232. 10.1037/h0048850 14472013

[B51] Metuki N. Sela T. Lavidor M. (2012). Enhancing cognitive control components of insight problems solving by anodal tDCS of the left dorsolateral prefrontal cortex. *Brain Stimul.* 5 110–115. 10.1016/j.brs.2012.03.002 22483547

[B52] Miniussi C. Harris J. A. Ruzzoli M. (2013). Modelling non-invasive brain stimulation in cognitive neuroscience. *Neurosci. Biobehav. Rev.* 37 1702–1712. 10.1016/j.neubiorev.2013.06.014 23827785

[B53] Moss F. Ward L. M. Sannita W. G. (2004). Stochastic resonance and sensory information processing: A tutorial and review of application. *Clin. Neurophysiol.* 115 267–281. 10.1016/j.clinph.2003.09.014 14744566

[B54] Murphy O. W. Hoy K. E. Wong D. Bailey N. W. Fitzgerald P. B. Segrave R. A. (2020). Transcranial random noise stimulation is more effective than transcranial direct current stimulation for enhancing working memory in healthy individuals: Behavioural and electrophysiological evidence. *Brain Stimul.* 13 1370–1380. 10.1016/j.brs.2020.07.001 32659482

[B55] Peña J. Sampedro A. Gómez-Gastiasoro A. Ibarretxe-Bilbao N. Zubiaurre-Elorza L. Aguiar C. (2021). The effect of changing the balance between right and left dorsolateral prefrontal cortex on different creativity tasks: A transcranial random noise stimulation study. *J. Creat. Behav.* 55 1–17. 10.1002/jocb.496

[B56] Peña J. Sampedro A. Ibarretxe-Bilbao N. Zubiaurre-Elorza L. Ojeda N. (2019). Improvement in creativity after transcranial random noise stimulation (tRNS) over the left dorsolateral prefrontal cortex. *Sci. Rep.* 9:7116. 10.1038/s41598-019-43626-4 31068654 PMC6506544

[B57] Peña J. Sampedro A. Ibarretxe-Bilbao N. Zubiaurre-Elorza L. Aizpurua A. Ojeda L. (2020). The effect of transcranial random noise stimulation (tRNS) over bilateral posterior parietal cortex on divergent and convergent thinking. *Sci. Rep.* 10:15559. 10.1038/s41598-020-72532-3 32968171 PMC7511964

[B58] Perrotta D. Bianco V. Berchicci M. Quinzi F. Perri R. L. (2021). Anodal tDCS over the dorsolateral prefrontal cortex reduces Stroop errors. A comparison of different tasks and designs. *Behav. Brain Res.* 405:113215. 10.1016/j.bbr.2021.113215 33662440

[B59] Pick H. Lavidor M. (2019). Modulation of automatic and creative features of the remote associates test by angular gyrus stimulation. *Neuropsychologia* 129 348–356. 10.1016/j.neuropsychologia.2019.04.010 31004692

[B60] Plucker J. A. Runco M. A. Simonsen M. A. (2020). “Enhancement of creativity,” in *The curated reference collection in neuroscience and biobehavioral psychology*, 3rd Edn, (Amsterdam: Elsevier Science Ltd), 10.1016/B978-0-12-809324-5.06181-2

[B61] Poreisz C. Boros K. Antal A. Paulus W. (2007). Safety aspects of transcranial direct current stimulation concerning healthy subjects and patients. *Brain Res. Bull.* 72 208–214. 10.1016/j.brainresbull.2007.01.004 17452283

[B62] Sellaro R. Hommel B. de Kwaadsteniet E. W. van de Groep S. Colzato L. S. (2014). Increasing interpersonal trust through divergent thinking. *Front. Psychol.* 5:561. 10.3389/fpsyg.2014.00561 24936194 PMC4047710

[B63] Sharma S. Babu N. (2017). Interplay between creativity, executive function and working memory in middle-aged and older adults. *Creat. Res. J.* 29 71–77. 10.1080/10400419.2017.1263512

[B64] Simonsmeier B. A. Grabner R. H. Hein J. Krenz U. Schneider M. (2018). Electrical brain stimulation (tES) improves learning more than performance: A meta-analysis. *Neurosci. Biobehav. Rev*. 84 171–181. 10.1016/j.neubiorev.2017.11.001 29128578

[B65] Snowball A. Tachtsidis I. Popescu T. Thompson J. Delazer M. Zamarian L. (2013). Long-term enhancement of brain function and cognition using cognitive training and brain stimulation. *Curr. Biol.* 23 987–992. 10.1016/j.cub.2013.04.045 23684971 PMC3675670

[B66] Sun J. Liu Z. Rolls E. T. Chen Q. Yao Y. Yang W. (2019). Verbal creativity correlates with the temporal variability of brain networks during the resting state. *Cereb. Cortex* 29 1047–1058. 10.1093/cercor/bhy010 29415253

[B67] Terney D. Chaieb L. Moliadze V. Antal A. Paulus W. (2008). Increasing human brain excitability by transcranial high-frequency random noise stimulation. *J. Neurosci.* 28 14147–14155. 10.1523/JNEUROSCI.4248-08.2008 19109497 PMC6671476

[B68] Torrance E. P. (1966). *The torrance tests of creative thinking-norms-technical manual research edition-verbal tests, forms a and b-figural tests, forms A and B.* Princeton, NJ: Personnel Press.

[B69] Tremblay D.-G. Pilati T. (2013). “Social innovation through arts and creativity,” in *The international handbook on social innovation* (Northampton, MA: Edward Elgar Publising Inc), 67–79. 10.4337/9781849809986.00015

[B70] van der Groen O. Wenderoth N. (2016). Transcranial random noise stimulation of visual cortex: Stochastic resonance enhances central mechanisms of perception. *J. Neurosci.* 36 5289–5298. 10.1523/JNEUROSCI.4519-15.2016 27170126 PMC6601807

[B71] Vanneste S. Fregni F. De Ridder D. (2013). Head-to-head comparison of transcranial random noise stimulation, transcranial AC stimulation, and transcranial DC stimulation for tinnitus. *Front. Psychiatry* 4:158. 10.3389/fpsyt.2013.00158 24391599 PMC3866637

[B72] Wan F. (2021). Statistical analysis of two arm randomized pre-post designs with one post-treatment measurement. *BMC Med. Res. Methodol.* 21:150. 10.1186/s12874-021-01323-9 34303343 PMC8305561

[B73] Ward L. M. (2009). Physics of neural synchronisation mediated by stochastic resonance. *Contemp. Phys.* 50 563–574. 10.1080/00107510902879246

[B74] Weinberger A. B. Green A. E. Chrysikou E. G. (2017). Using transcranial direct current stimulation to enhance creative cognition: Interactions between task, polarity, and stimulation site. *Front. Hum. Neurosci.* 11:246. 10.3389/FNHUM.2017.00246 28559804 PMC5432551

[B75] Wilcox A. J. Cortese M. Baravelli C. M. Skjaerven R. (2018). When intuition invites the analytical mind to dance-the essential role of creativity in science. *Epidemiology* 29 753–755. 10.1097/EDE.0000000000000913 30074539 PMC6193552

[B76] Xuejun B. Yanbin G. Weiping H. Qing H. Haijun Y. (2014). The inhibitory mechanism of individuals with different scientific creativity. *Stud. Psychol. Behav.* 2:151.

[B77] Yamada Y. Nagai M. (2015). Positive mood enhances divergent but not convergent thinking. *Jpn. Psychol. Res.* 57 281–287. 10.1111/jpr.12093

[B78] Zhang W. Sjoerds Z. Hommel B. (2020). Metacontrol of human creativity: The neurocognitive mechanisms of convergent and divergent thinking. *NeuroImage* 210:16572. 10.1016/j.neuroimage.2020.116572 31972282

[B79] Zmigrod S. Colzato L. S. Hommel B. (2015). Stimulating creativity: Modulation of convergent and divergent thinking by transcranial direct current stimulation (tDCS). *Creat. Res. J.* 27 353–360. 10.1080/10400419.2015.1087280

